# Lactate-Dependent Regulation of Immune Responses by Dendritic Cells and Macrophages

**DOI:** 10.3389/fimmu.2021.691134

**Published:** 2021-07-29

**Authors:** Indumathi Manoharan, Puttur D. Prasad, Muthusamy Thangaraju, Santhakumar Manicassamy

**Affiliations:** ^1^Georgia Cancer Center, Medical College of Georgia, Augusta University, Augusta, GA, United States; ^2^Department of Biochemistry and Molecular Biology, Medical College of Georgia, Augusta University, Augusta, GA, United States; ^3^Department of Medicine, Medical College of Georgia, Augusta University, Augusta, GA, United States

**Keywords:** dendritic cells, macrophages, lactate signaling, GPR81/GPR132, regulatory and inflammatory responses, antitumor immunity, immune response to infections, inflammatory diseases

## Abstract

For decades, lactate has been considered an innocuous bystander metabolite of cellular metabolism. However, emerging studies show that lactate acts as a complex immunomodulatory molecule that controls innate and adaptive immune cells’ effector functions. Thus, recent advances point to lactate as an essential and novel signaling molecule that shapes innate and adaptive immune responses in the intestine and systemic sites. Here, we review these recent advances in the context of the pleiotropic effects of lactate in regulating diverse functions of immune cells in the tissue microenvironment and under pathological conditions.

## Introduction

For decades, lactate has been considered a waste product of cellular metabolism. New lines of investigations now recognize this glycolytic metabolite as an active immune modulator that shapes the functions of immune cells in the tissue microenvironment and under pathological conditions (1–3). Accumulation of lactate in the tissue microenvironment is an essential feature of both inflammatory diseases and cancer (1–3). Much is known about the production of lactic acid under various disease conditions, while the mechanisms by which it shapes the effector functions of immune cells and restores tissue homeostasis remain obscure. Recent evidence suggests an emerging role for lactate in the field of inflammation, autoimmunity, and cancer (1–3). Here, we review our current knowledge of the role of lactate in regulating inflammatory and regulatory responses in various tissue environments, highlight some unanswered questions, and discuss how this new information can be exploited in the rational design of therapies against various autoimmune disorders, infections, and cancers.

## Dendritic Cells and Macrophages in Regulating Inflammatory and Regulatory Responses

The immune system launches robust immunity against foreign antigens while maintaining a state of tolerance to self-antigens, food antigens, and commensal flora (4–8). Loss of tolerance to self-antigens, food antigens, and commensal flora leads to immune cell-mediated inflammatory diseases and autoimmunity (4–9). Dendritic cells (DCs) and macrophages are a specialized subset of antigen-presenting cells (APCs) that form a critical link between innate and adaptive immune cells. These APCs represent a complex immunological system composed of several functionally distinct subsets distributed in different organs and microenvironments (4–9). A detailed discussion of DC and macrophage subsets and their influence on adaptive immunity is outside the scope of the present review and was reviewed extensively recently (4–9). DCs and macrophages express a wide array of pathogen recognition receptors (PRRs) which enables them to sense different pathogen-associated molecular patterns (PAMPs) and damage‐associated molecular pattern molecules (DAMPs) (7, 10). Signaling through PRRs activate and program APCs to induce distinct innate responses that shape the type of T-helper (Th) responses (7, 10). In addition to inducing robust immune responses against infections, DCs and macrophages also play a critical role in suppressing inflammatory responses and maintaining tissue immune homeostasis. Furthermore, DCs and macrophages induce immune tolerance and contribute to the resolution of inflammation through several regulatory mechanisms (11). However, the cellular and molecular mechanisms underlying this phenomenon remain poorly understood. Emerging studies suggest a fundamental role for lactate in the tissue microenvironment in regulating immunity and immune tolerance by shaping the functions of DCs and macrophages. Here, we will discuss how lactate shapes the functions of DCs and macrophages under steady-state and inflammatory conditions.

## Lactate Metabolism and Transport in Dendritic Cells and Macrophages

Upon activation, DCs and macrophages undergo profound metabolic changes critical for biosynthesis and energy production (12). Lactate could also serve as a fuel source to produce energy by various cell types, including immune, cancer, and stromal cells (13, 14). Like other cell types, APCs produce lactate under hypoxic conditions or by aerobic glycolysis. A phenomenon similar to the Warburg effect in tumors is also observed in DCs and macrophages following TLR activation which induces a major metabolic reprogramming characterized by a switch from oxidative phosphorylation (OXPHOS) to glycolysis. This metabolic shift reprograms APCs from a regulatory state to an inflammatory state and intracellular and extracellular lactate levels play an essential role in this process.

Under homeostatic conditions, intracellular and extracellular lactate levels are tightly regulated. Lactate production occurs in the cytoplasm within the cell due to hypoxic or aerobic glycolysis and accumulates in the extracellular space. Lactate dehydrogenases (LDHs) are critical enzymes in glycolysis that reversibly catalyze the conversion of pyruvate to lactate or lactate to pyruvate (1, 13). LDH is a tetrameric enzyme composed of two types of subunits namely LDH-A and LDH-B. LDH-A has a higher affinity for pyruvate and preferentially catalyzes pyruvate to L-lactate, while LDH-B has a higher affinity for lactate and converts L-lactate to pyruvate, fueling oxidative metabolism. Immune cells, including DCs and macrophages, express both LDH-A and LDH-B subunits (1). However, proinflammatory DCs and macrophages express higher levels of LDH-A and show increased production of lactate due to the sustained glycolytic reprogramming induced by TLR ligands. In DCs this metabolic shift depends on activating transcription factors such as sterol regulatory element-binding protein (SREBP) and hypoxia-inducible factor (HIF)-1α (15). Furthermore, HIF-1α plays a crucial role in regulating the expression of LDH-A and several other genes involved in glycolysis (15). Evidence suggests that HIF1α deficiency in DCs and macrophages leads to loss of GLUT1 (a facilitative glucose transporter) and LDHA (16–18). Besides, HIF-1α can be activated through a feedback mechanism by intracellular pyruvate or lactate (19, 20). However, whether this effect depends on SREBP or the direct control of inflammatory cytokine expression is unknown.

Lactate in the cell or extracellular space is transported across the plasma membrane by monocarboxylate transporters (MCTs) of the SLC16 solute carrier family, and they transport lactate by an H^+^-coupled transport mechanism (13, 14, 21, 22). MCTs, prevent intracellular accumulation of lactate by removing excess lactate produced due to increased glycolytic activity (23, 24). Dendritic cells and macrophages express MCT1, MCT2 and MCT4 (13, 14, 22). MCT1 and MCT2 have a higher affinity for lactate and are primarily responsible for transporting lactate into the cells. MCT4 has a lower affinity for lactate and is primarily responsible for the export of lactate. Interestingly, lactate also regulates the expression of MCT1 and MCT4 (25). In addition to MCTs, two other solute carrier family 5 members (SLC5), namely SLC5A8 (SMCT1, sodium-coupled monocarboxylate transporter 1) and SLC5A12 (SMCT2, sodium-coupled monocarboxylate transporter 2) can also mediate transmembrane transfer of lactate, and they transport lactate by a Na^+^-coupled transport mechanism. DCs, macrophages, and other immune cells express SLC5A8 and SLC5A12 (13, 14, 22). MCTs and SMCTs play a key role in lactate transport in APCs, yet their regulation and roles under steady-state and inflammatory conditions are incompletely understood.

### The Lactate-Mediated Signaling Pathway

New lines of investigation now place lactate as an active signaling molecule that controls the differentiation and functions of immune cells under steady-state and inflammatory conditions. In addition, lactate exerts autocrine effects on the host cells and paracrine effects on other cell types in the tissue environment. Recent studies have revealed some of the signaling pathways by which lactate shapes the functions of DC and macrophage through receptor-dependent and receptor-independent mechanisms.

## Lactate-GPR81 Signaling Axis

**Figure 1:**
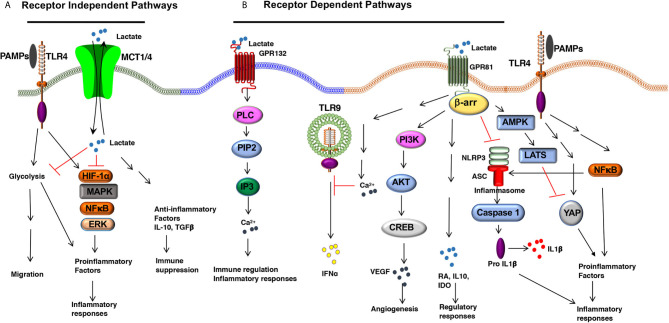
The Lactate-mediated receptor-dependent and receptor-independent signaling pathways. Lactate binds to GPR81 and GPR132 receptors and activates several downstream signaling pathways and transcription factors in DCs and macrophages. **(A)** Lactate binding to GPR81 and GPR132 results in the activation or suppression of several downstream pathways such as PI3K/AKT/CREB, PLC/IP3/Ca^2+^, β-arrestin/inflammasome, AMPK/LATS/YAP/NF-κB. This results in reduced expression of proinflammatory cytokine production and increased expression of immune regulatory factors (IL-10, IDO, RA, TGFβ) in response to TLR ligands. **(B)** Lactate can shape APC functions independent of surface receptors. MCTs transport extracellular lactate into the cells, and intracellular lactate can modulate APC functions by directly regulating the activation of multiple signaling pathways and transcription factors such as HIF-1α, MAPK, ERK, and NF-κB.

L-Lactate, a ubiquitous metabolite, functions as a natural ligand for GPR81 (HCAR1, hydroxy-carboxylic acid receptor) (26, 27). Lactate activates GPR81 in its physiological concentration range of 1–20 mM (17). GPR81 expression varies depending on the cell type and tissue microenvironment. For example, fat cells express high levels of GPR81 whereas secondary lymphoid tissues, gut, brain, kidney express low levels of GPR81 (26, 28, 29). Recently, several groups have reported that DCs and macrophages express GPR81, and its expression is regulated by the tissue microenvironment (29–32). Our recent work has shown that DCs and macrophages in the intestine and lung express higher levels of GPR81 compared with DCs and macrophages in the spleen (30). Likewise, DCs in the tumor microenvironment (TME) express high levels of GPR81 (13, 32). An important unresolved question is how the tissue microenvironment regulates GPR81 expression in these APCs. Adipocyte studies have shown that peroxisome proliferative–activated receptor γ (PPARγ) transcriptionally regulates GPR81 expression (27, 33). Lipids and their metabolites are potent activators of the PPAR family transcription factors in APCs (34–36). These ligands are widely present in the intestine and TME suggesting that PPAR-mediated signaling might regulate GPR81 expression in APCs (34–36). Besides, recent studies have shown that lactate can regulate GPR81 expression in tumor cells *via* the snail3/STAT3 (signal transducer and activator of transcription 3) pathway (37). Further studies are warranted to see whether the PPARγ and snail3/STAT3 pathways regulate GPR81 expression in DCs and macrophages. Recent studies have highlighted a protective role for GPR81 in minimizing tissue injury by controlling pathological inflammatory responses (31). Lactate-GPR81 mediated signaling in non-immune cells regulates several key signaling pathways such as the cyclic adenosine monophosphate (cAMP), protein kinase A (PKA), and extracellular signal-regulated kinase (ERK) pathways. However, the downstream signaling networks of GPR81 in DCs and macrophages are unknown. GPR81 suppresses inflammatory responses in monocytes and macrophages by limiting the activation of the β-arrestin/inflammasome pathway (31). In pDCs, GPR81 signaling regulates IFNα production by inducing intracellular Ca^2+^ mobilization and its downstream genes Ca^2+^/calmodulin dependent protein kinase II (CaMKII), and calcineurin (CaN) phosphatase (38). In addition to modulating these pathways, other signaling pathways, such as inhibition of nuclear factor-kappa B (NF-κB), play a role in the anti-inflammatory function of lactate in macrophages. GPR81 signaling in macrophages exerts suppressive effects on NF-κB and yes-associated protein (YAP) activation *via* activation of AMP-activated protein kinase (AMPK) and large tumor suppressor kinases (LATS), resulting in reduced proinflammatory cytokine production after exposure to LPS (39) (**Figure 1**). In contrast to its anti-inflammatory role, an *in vitro* study has shown that lactate augmented LPS-induced expression of inflammatory genes by enhancing NF-κB activation in human monocyte-derived macrophages and U937 cells (40). In the TME, GPR81-signaling plays an essential role in immune suppression against tumors by inducing regulatory APCs (32) and upregulating the expression of programmed death-ligand 1 (PD-L1) in tumor cells (25). Collectively, these studies show a regulatory role for the lactate-GPR81 signaling axis in DCs and macrophages. GPR81 signaling in tumor cells regulates MCT1 and MCT4 (25), but underlying molecular mechanisms remain largely unknown. The extent to which GPR81 signaling regulates the expression of MCT1 and MCT4 in DCs and macrophages remains to be determined.

## Lactate-GPR132 Signaling Axis

A second functional receptor for lactate in macrophages is the G protein-coupled receptor 132 (Gpr132, also known as G2A) (41, 42). Besides lactate, lysophosphatidylcholine (lysoPC) is also a ligand for GPR132 (43). GPR132 is a stress-inducible, seven-pass transmembrane receptor that actively modulates several cellular and biological activities, such as cell cycle, proliferation, and immunity (44, 45). Tumor-associated macrophages (TAMs) promote metastasis (46, 47), and GPR132 signaling plays a crucial role in promoting breast cancer metastasis (41). Mechanistically, the lactate-GPR132 axis promotes the alternatively activated macrophage (M2)-like phenotype, which, in turn, facilitates cancer cell adhesion, migration, and invasion (41). Consequently, GPR132 deletion reduces M2 macrophages and impedes breast cancer lung metastasis in mice. Interestingly, GPR132 regulating macrophage function may vary depending on the tissue microenvironment (41, 42). Like GPR81, GPR132 plays a crucial role in regulating inflammation in the intestine (48). GPR132-mediated signaling activates several downstream signaling pathways associated with immune regulation and inflammatory responses such as cAMP, protein kinase A (PKA), and ERK (48) (**Figure 1**). However, the role of GPR132 in intestinal macrophages and DCs remain largely unknown.

## Receptor-Independent Lactate Signaling

In addition to signaling *via* cell surface receptors, extracellular lactate can also modulate the APC’s functions by directly regulating the activation of multiple signaling pathways and transcription factors after getting transported into the cells through MCTs and SMCTs (14). In this context, ex vivo studies have shown that lactate metabolically reprograms macrophages to inhibit the expression of proinflammatory factors in response to LPS in a GPR81-independent manner (29). Under hypoxic conditions, lactate can modulate DC and macrophage functions by regulating other signaling pathways such as the HIF-1α, Hedgehog, MAPK, and mTOR pathways (49) (**Figure 1**). Histone deacetylases (HDACs) regulate gene transcription and chromatin assembly at the posttranscriptional levels by modifying histones (50). HDAC inhibitors exhibited anti-inflammatory effects and were shown to ameliorate immune cell-mediated inflammatory diseases (50). For instance, strong evidence shows that intracellular lactate acts as an endogenous inhibitor of HDACs and regulates gene transcription in an HDAC-dependent manner (51, 52). Further, studies are warranted to see whether lactate regulates gene transcription in DCs and macrophages *via* the inhibition of HDACs.

## Modulation of DC and Macrophage Functions by Lactate

**Figure 2:**
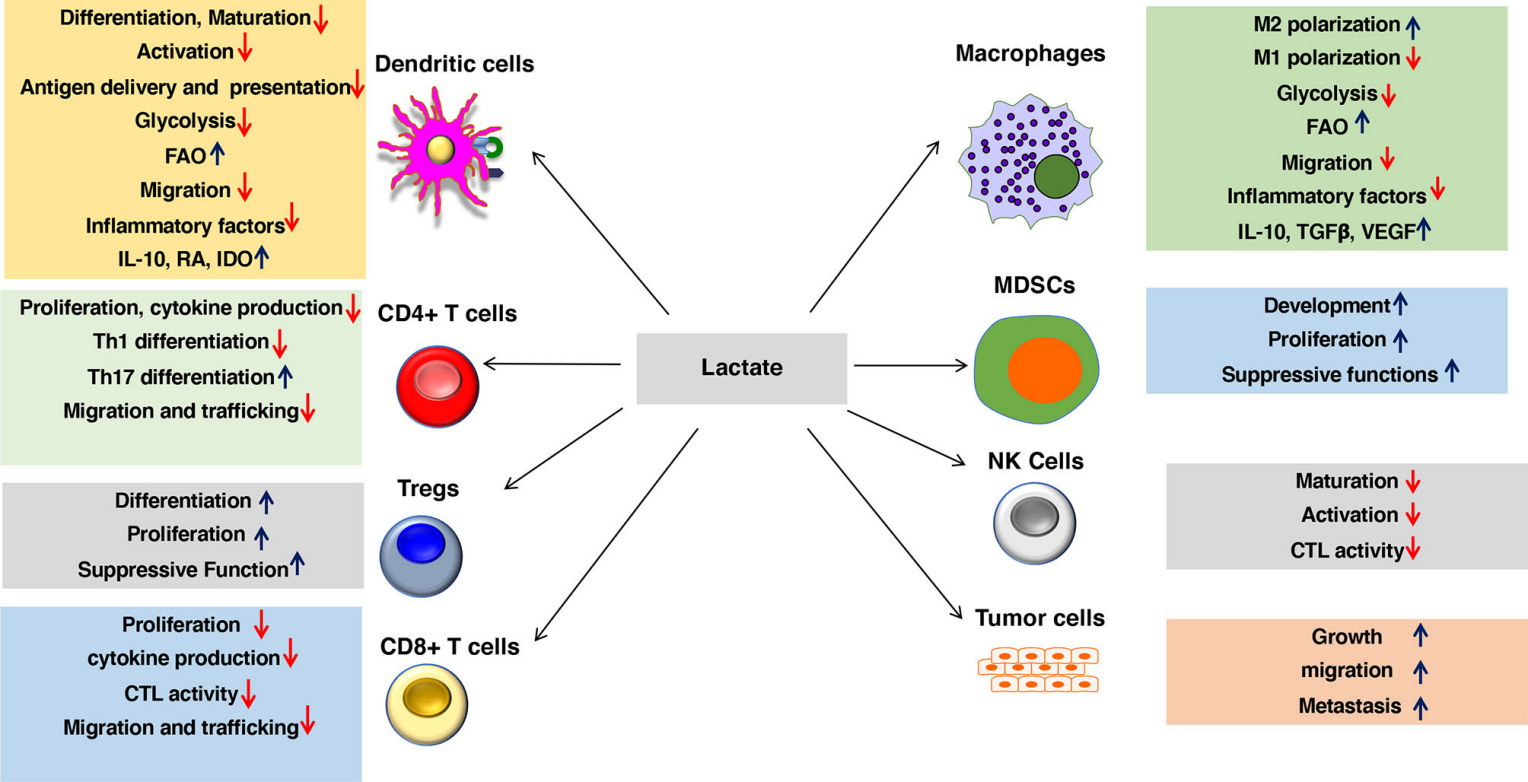
Lactate in the environment shapes the functions of both innate and adaptive immune cells. Lactate promotes anti-inflammatory and antitumor immune responses by modulating DC and macrophage functions such as activation, trafficking, capturing, and cross-presenting antigens and expression of immune regulatory and inflammatory factors. Besides, lactate signaling cascade directly shapes the activation, proliferation, and effector phenotypes of myeloid-derived suppressor cells (MDSCs), Tregs, CD4^+^ T cells, CD8^+^ T cells, NK cells, and other immune cells. Lactate also regulates trafficking and migration of immune cells to tissues and draining lymph nodes (DLNs) by regulating chemokine receptors as well as synthesis and secretion of chemokines. Lactate signaling in the immune cell leads to metabolic alterations in DCs and macrophages that programs them to a regulatory state. Lactate-mediated signaling shifts metabolism of DCs and macrophages from glycolysis to fatty acid oxidation (FAO). Besides, lactate signaling in tumors and macrophages promotes tumor growth, migration, and metastasis.

Robust immune responses against pathogens and tumors depend on several factors, such as the degree of maturation and activation of DCs, their ability to capture, process, and present exogenous antigens, them trafficking to secondary lymphoid organs and tissues type of factors they produce. Emerging studies have shown that lactate-mediated signaling is crucial in shaping immune responses by modulating DC and macrophage functions (**Figure 2** and **Table 1**). Here, we will discuss how lactate shapes essential functions of DCs and macrophages that influence adaptive immune responses.

**Table 1:** Evidence for involvement of the lactate in the microenvironment in shaping the functions of innate and adaptive immune cells.

Observations	References
**Lactate effects on DC function**	
Lactate suppresses DC differentiation and maturation.	(53–59)
Lactate suppresses the activation of DCs and the expression of proinflammatory factors in response to TLR ligands.	(31, 53)
Lactate inhibits antigen delivery and presentation by DCs.	(53, 60–62)
Lactate accelerates antigen degradation in DCs by downregulating membrane trafficking proteins.	(57)
Lactate-GPR81 signaling in intestinal DCs induces the expression of immune regulatory factors to induce Tregs and suppresses the differentiation of Th1/Th17 cells.	(30)
Lactate in the TME conditions DCs to a regulatory state to suppress antitumor immune responses.	(53, 60–62)
Tumor DCs-deficient in GPR81 are more potent in inducing antitumor immunity.	(32)
Lactate signaling regulates the expression of chemokine receptors and chemokines that are critical for DC migration.	(53, 63–65)
Lactate signaling in DCs regulates metabolic pathways involving glycolysis and fatty acid oxidation (FAO)	(60, 66)
**Lactate effects on T cells**	
Lactate suppresses T cell proliferation, cytokine production and Th1 differentiation.	(67–69)
Lactate promotes Treg proliferation and functions.	(67, 70–72)
Under inflammatory conditions, lactate signaling in CD4^+^ T cells favors Th17 cell differentiation.	(73, 74)
Lactate suppresses the T cell migration and trafficking.	(73, 74)
Tumor-derived lactate limits the expansion of tumor-antigen specific CD8^+^ T cells, cytokine production, CTL activity.	(75, 76)
Lactate synergizes with IL-21 to promote stemness of CD8^+^ T cells and antitumor immunity.	(70)
**Lactate effects on macrophage**	
Lactate induces alternative polarization (M2) of macrophages.	(29, 41, 77)
Lactate signaling in macrophages attenuates TLR-induced proinflammatory cytokine production.	(31, 39, 59)
Lactate signaling in TAMs promotes tumor growth, migration, metastasis, and immunosuppression.	(20, 41, 78–80) (41, 80, 81)
Lactate-GPR132 signaling in macrophages contributes to tumor cell invasiveness and tumor growth.	(65)
Lactate-GPR81 signaling imparts regulatory phenotype on intestinal macrophages and induces the expression of immune regulatory factors to induce Tregs.	(30)
Lactate-GPR81 signaling in macrophages suppresses expression of inflammatory factors in response to LPS.	(31, 39)
**Lactate effects on MDSCs**	
Lactate promotes the development and accumulation of MDSCs in tumors.	(82)
Lactate-conditioned MDSCs inhibit the function of natural killer (NK) cells and T lymphocytes	(83, 84)
**Lactate effects on NK cells**	
Tumor-derived lactic acid inhibits natural killer (NK) cell maturation and function.	(75)
**Lactate effects on other immune cells**	
Lactate regulates the functions of basophils, neutrophils, mast cells	(85–88)
**Lactate effects on Tumor cells**	
Lactate promotes tumor growth, migration, and metastasis,	(13, 89, 90)

## Regulation of DC Maturation and Activation by Lactate

DC maturation and activation are important in inducing a robust immune response against tumors and pathogens (11). Immature or tolerogenic DCs facilitate tolerance or immune regulatory responses, whereas immunogenic/inflammatory DCs facilitate robust inflammatory responses (7). Under homeostatic conditions, peripheral DCs typically display an immature phenotype characterized by low surface levels of MHC II and costimulatory molecules and induce suboptimal T‐cell priming, often leading to T‐cell anergy or tolerance. Upon stimulation, DCs undergo maturation characterized by the expression of high levels of MHC II and costimulatory molecules (CD80, CD86, and CD40) and induce robust T‐cell activation and effector differentiation (7). However, certain stimuli induce the tolerogenic/regulatory DCs that express markedly lower costimulatory molecules and induce regulatory T cells and immune suppression. Several reports have shown that lactate-mediated signaling blocks DC differentiation, activation and antigen presentation (53, 60). Exposure to lactate conditions DCs to a regulatory or anti-inflammatory state. Earlier *ex vivo* studies have shown that DCs cultured in the presence of lactate exhibit regulatory functions (53–59). These regulatory DCs expressed low surface levels of MHC II and costimulatory molecules and produced markedly lower levels of IL-12 and higher levels of IL-10. Besides, a recent study has shown that human tolerogenic DCs produce high levels of lactate that shape T cell responses toward tolerance and delayed graft-versus-host disease (91). Like DCs, lactate exposure polarizes macrophages to M2 phenotype with increased expression of CD163 and Arg1 and decreased expression of M1 markers such as CD38, iNOS, IL-1β, and IL-6 (41). This lactate effect on TAMs depends on NRF2 (nuclear factor erythroid 2-related factor 2), HIF-1α, and MCT1 (20, 78, 79).

DCs and macrophages recognize diverse microbial structures through multiple receptors collectively known as PRRs (10). DCs and macrophages can also recognize damage-associated molecular patterns (DAMPs) and other endogenous ligands released from dying tumor cells through PPRs (92–94). PRRs include Toll-like receptors (TLRs), C-type lectin-like receptors (CLRs), RIG-I-like receptors (RLRs), and Nod-like receptors (NLRs) (10). TLR ligands have gained significant interest in immunotherapy in recent years for their potential use as vaccine adjuvants (95, 96). In general, PRR engagement potently activates DCs by upregulating the surface expression of maturation markers such as MHCII, CD80, CD83, and CD86 (10). Even though PRR ligands are there in the TME and mucosal organs (92–94), DCs and macrophages present in these environments display markedly decreased expression of costimulatory molecules (97). Earlier *ex vivo* studies on human and murine DCs have shown that exposure to lactate markedly affected the maturation and activation in response to LPS (31, 53). Lactate also inhibited the LPS-mediated activation of bone marrow-derived macrophages and peritoneal macrophages. Lactate-conditioned macrophages failed to upregulate costimulatory molecules while expressing lower levels of proinflammatory cytokines and higher levels of IL-10 even in response to TLR ligands (29, 31, 39, 98). Further, mechanistic studies have shown that lactate signaling can negatively regulate the inflammatory pathways such as the NF-kB, NFAT (nuclear factor of activated T-cells), YAP, inflammasome, and MAPK (mitogen-activated protein kinases) pathways, critical for DC activation and expression of inflammatory factors (31, 59). Accordingly, DCs and macrophages that are deficient in GPR81 are hyper-responsive to TLR ligands (30). Also, other studies revealed the role of monocarboxylate transporters (MCTs) in mediating the lactate effect in macrophages (77). MCT4 inhibition significantly boosted lactate-induced M2 polarization, while blocking of MCT1/2 failed to reverse the immunosuppressive effect of lactate, correlating with the results from gene expression studies that showed lactate increasing MCT4 expression but downregulating the expression of MCT1/2 (59). Thus, the effects of lactate on the maturation and activation of DCs and macrophages involve GPR81 dependent and independent mechanisms *via* MCTs.

## Regulation of Dendritic Cell Migration by Lactate

The migration of DCs to secondary lymphoid organs and tissues is essential for initiating adaptive immune responses, tumor immune surveillance, regulation of inflammation in the tissues, and selective elimination of infected cells (99, 100). DC migration involves its trafficking to tissues, capturing and endocytosing dead or infected cells, and transporting associated antigens to the draining lymph nodes (TDLNs) where they prime and activate T cells to initiate adaptive immune responses (101–104). DC migration depends on the expression of specific chemokine receptors on DCs and its cognate chemokine ligand within the tissues and DLNs. DC migration to DLNs requires chemokine receptor CCR7, whereas its recruitment to the tissues depends on chemokines such as CCL4, CCL5, and XCL1 (99). However, only a tiny fraction of DCs migrate to tumor tissue and subsequently to the draining lymph nodes. Glycolytic metabolism is essential for CCR7 oligomerization and DC migration (94). Blocking glycolysis impairs CCR7 oligomerization and impairs migration (63). Ex vivo studies have shown that high lactate levels (20 mM) inhibited the migration of monocytes and DCs (53, 64). Similarly, lactate regulates macrophage functions such as adhesion, migration, and tissue recruitment (41, 80, 81). Furthermore, lactate in the TME and inflamed tissues can regulate the migration of immune cells by regulating the expression of several key enzymes involved in glycolysis (105).

## Regulation of Antigen Delivery and Presentation by Lactate

Cross-presentation is critical for initiating immune responses against tumors and viral infections, where DCs present extracellular antigens on MHC I to activate CD8^+^ T cell-mediated cytotoxicity (10). Effective cross-presenting involves uptake of extracellular antigens, processing antigens into peptides, loading peptides onto MHC I, and trafficking of MHC I: peptide complex to the cell’s surface (106). Membrane trafficking proteins such as SNARE (soluble n-ethylmaleimide-sensitive factor attachment protein receptor) and VAMP3 (vesicle-associated membrane protein 3) play a critical role in cross-presentation. Loss of these membrane trafficking proteins in DCs leads to defective cross-presentation of tumor-associated antigens (57). DCs within the TME are less efficient in cross-priming CD8^+^ T cells (61, 62), and the TME contains high lactate levels. Emerging evidence has shown that lactate affects DCs’ function by regulating antigen presentation and cross-priming CD8^+^ T cells (53, 60). However, the underlying molecular mechanisms by which lactate affects cross-presentation are not known. In this context, a recent study has shown that lactate can affect cross-presentation by downregulating membrane trafficking proteins such as SNAREs and VAMP3 while accelerating antigen degradation in DCs (57). Furthermore, these proteins facilitate the secretion of cytokines from DCs upon activation. Further studies are warranted to see whether the regulation of membrane trafficking proteins by lactate is dependent on GPR81 and MCTs. These studies collectively suggest that the lactate-mediated signaling suppresses efficient capture of tumor-associated antigens by tumor DCs and cross-priming of CD8^+^ T cells.

## Regulation of Immune Regulatory and Inflammatory Factors by Lactate

DCs dictate the fate of naïve CD4^+^ and CD8^+^ T cells through differential production of pro- and anti-inflammatory cytokines (11, 107). Recent studies have shown that lactate can shape the adaptive immune responses by regulating the expression of immune regulatory factors and inflammatory factors in DCs and macrophages (108–113). DCs and macrophages exposed to TLR-ligands produce markedly higher levels of proinflammatory cytokines and type-I interferons (IFN). In contrast, lactate-conditioned DCs and macrophages do not release immunostimulatory cytokines; instead, they express higher levels of IL-10 in response to TLR ligands (53–55, 57, 58). The TME contains higher levels of immune regulatory factors such as IL-10, retinoic acid (RA), and TGF-β that actively suppress differentiation and expansion of tumor-specific effector T cells (114, 115). Lactate in the TME condition DCs and macrophages to a regulatory or anti-inflammatory state (1, 2). Accordingly, tumor DCs deficient in the lactate receptor GPR81 expressed markedly higher levels of IL-12 and IL-6 (32). Similarly, lactate-GPR81 signaling influences the pDC functions in tumors by attenuating IFNα production (38). Furthermore, blocking GPR81 signaling can restore the IFNα production by pDCs. Lactate in the TME conditions DCs and macrophages to express higher levels of IL-10 (1, 2). The effects of lactate on the expression of the regulatory and inflammatory cytokines in APCs also depend on MCTs. Furthermore, blocking the MCT in DCs or macrophages can reprogram them to an inflammatory state (59). These APCs produce high levels of inflammatory factors in response to TLR ligands (38). In the intestine, anti-inflammatory factors such as IL‐10, TGF‐β, IDO, and RA produced by DCs and macrophages are critical for maintaining immune tolerance to commensal flora (116, 117). These immune regulatory factors are also necessary to suppress inflammation and restore immune homeostasis in the intestine. A recent study has highlighted an essential role for the lactate-GPR81 signaling in intestinal DCs and macrophages in regulating the expression of immune regulatory factors such as IL-10, retinoic acid (RA), and IDO (30). Intestinal DCs and macrophages isolated from GPR81 deficient mice produced markedly higher levels of inflammatory cytokines and lower levels of anti-inflammatory factors under homeostatic and inflammatory conditions (30). Furthermore, GPR81-deficient intestinal APCs are hyper-responsive to microbial ligands and express higher levels of proinflammatory cytokines (30). Collectively, these studies demonstrate that lactate-mediated signaling imparts an anti-inflammatory phenotype to DCs and macrophages.

## Regulation of Immune Cell Metabolism by Lactate

Cellular metabolic pathways play a critical role in modulating the functions of DCs and macrophages (12, 56), and emerging evidence support lactate as one of the essential molecules that links metabolism and immunity. DC and macrophage subsets have potential metabolic differences under homeostatic and inflammatory conditions (12, 56). Tolerogenic or regulatory DCs and macrophages show a catabolic metabolism marked by increased oxidative phosphorylation, fatty acid oxidation (FAO), and glutaminolysis (12, 56). In contrast, immunogenic or inflammatory DCs display an anabolic metabolism marked by increased glycolysis and lactate production (12, 56). Preliminary evidence suggests that lactate mediates immune cell-intrinsic effects on metabolism (73). Besides, extracellular lactate induces metabolic reprogramming of DCs and macrophages, resulting in reduced glycolysis and increased FAO (29, 60, 66). This metabolic reprogramming of APCs significantly changes cytokine production with predominantly anti-inflammatory effects, emphasizing the complex interplay between metabolism and APC functions (29, 60, 66). The effects of lactate on immune cell metabolism may serve as a negative feedback signal limiting inflammation (3). For example, lactate can modulate APC functions by regulating the expression of critical enzymes involved in glycolysis (3). These studies show that lactate imparts regulatory phenotype on APCs by metabolic reprogramming.

## Effects of Lactate in Modulating the Functions of Other Immune Cells

Emerging studies are beginning to provide insights into the mechanisms by which lactate signaling cascade directly shapes the effector phenotypes of myeloid-derived suppressor cells (MDSCs), Tregs, CD4^+^ T cells, CD8^+^ T cells, and natural killer (NK) cells. Several excellent studies and reviews discuss extensively how extracellular lactate shapes the functions of other immune cells (12, 56) and will thus be discussed only briefly (**Table 1**). Lactate can exhibit a proinflammatory or anti-inflammatory effect depending on the microenvironment and immune cell type conditions and factors. For example, lactate exerts an immune-suppressive role in the TME, whereas lactate exerts an inflammatory role in chronic conditions like arthritis. Lactate in the TME promotes expansion and accumulation of MDSCs while suppressing the effector functions of NK cells, CD4^+^ T lymphocytes, CTLs and mast cells (83–85). On the other hand, under chronic inflammatory conditions, lactate manifests an inflammatory role on CD4^+^ T cells by promoting the differentiation of Th17 cells (73). Effect of lactate on Th17 cell differentiation is T cell-intrinsic, but the underlying molecular mechanism is unknown and requires further investigation. These studies collectively show that the multi-faceted effect of lactate on the immune response is dependent on cellular and environmental contexts.

## Regulation of Autoimmunity and Antitumor Immunity by Lactate

Accumulation of lactate in the tissue microenvironment is a feature of both inflammatory disease and cancer. Emerging evidence suggests that this is due to metabolic disturbances in immune cells. Lactate exhibits an inflammatory or anti-inflammatory role depending on its effects on immune cell type, disease type, and tissue environment (12, 56). This section will review recent developments in our understanding of the role of lactate-mediated signaling in regulating immune responses in pathological conditions.

## Inflammatory Bowel Disease (IBD)

Loss of immune tolerance to intestinal commensal flora and oral antigens leads to chronic intestinal inflammation and inflammatory bowel disease (IBD). In the colon, lactate is one of the primary metabolites produced by bacterial fermentation of dietary products and gastrointestinal mucosa is exposed to high concentrations of lactate (66, 73, 117). Besides, intestinal epithelial cells and immune cells can produce lactate (118–120). Initial study on murine models of IBD showed that the intrarectal treatment with lactate prevents intestinal inflammation by downregulating proinflammatory response in epithelial cells (121). However, whether lactate regulates immune responses to gut commensal flora remains largely unknown. Our recent work has revealed an essential role for GPR81 in programming tolerogenic DCs and macrophages in the intestine (30). Mice deficient in GPR81 are highly susceptible to chemically-induced colitis and T cell-mediated colitis. Besides, genetic deletion of GPR81 in mice led to loss of immune homeostasis in the intestine, which enhanced susceptibility to colonic inflammation (30). Besides intestinal APCs, lactate plays a crucial role in intestinal stem-cell-mediated regeneration of the epithelial layer through the GPR81-Wnt signaling pathway (122). This observation is particularly relevant in the intestine, given the importance of Wnt signaling in intestinal DCs and macrophages in regulating immune tolerance and commensal homeostasis in the intestine (123, 124). It would be interesting to see how lactate and Wnt signaling pathways cross-regulate each other in establishing immune tolerance and commensal homeostasis in the gut. In a striking functional similarity with GPR81 knockout mice, genetic deficiency of GPR132 also resulted in significantly worsened chemically-induced colitis in mice (48, 125). However, GPR132 deficiency does not alter intestinal immune homeostasis under homeostatic conditions. GPR132-mediated signaling in myeloid and lymphoid cells limits intestinal inflammation in a mouse model of colitis induced by dextran sodium sulfate (125). Collectively, these studies have identified a new and essential role for lactate, GPR81, and GPR132 signaling pathways in regulating immune tolerance and colonic inflammation.

## Other Immune-Mediated Inflammatory Diseases

Lactate plays a protective role in murine models of immune hepatitis and pancreatitis (31). In this model, the lactate-mediated protective effect is dependent on GPR81 signaling that limits the expression of proinflammatory factors by macrophages. Mice deficient in GPR81 are highly susceptible to LPS-induced hepatitis and pancreatitis. Confirming this finding, pharmacological activation of GPR81 decreased LPS-induced activation of the caspase-1 and NF-κB pathways and production of proinflammatory factors by macrophages and reduced disease severity in mice (31). Lactate plays a similar anti-inflammatory role in Multiple sclerosis (MS). MS is a chronic inflammatory demyelinating neurological disease of the central nervous system (CNS). In the experimental autoimmune encephalomyelitis (EAE) model of MS, macrophages in the CNS display higher expression of LDHA and increased glycolysis (126). CNS macrophages also expressed higher levels of MCT4. siRNA-mediated knockdown of LDHA and MCT4 or blocking MCT4 reduced leukocyte infiltration and the clinical severity of EAE (126). However, the effects of lactate on the functions of DCs and other immune cells in this chronic inflammatory disease are not known.

In contrast to its regulatory and protective role, lactate significantly induces and promotes inflammation in rheumatoid arthritis (RA) (1). RA is a chronic inflammatory disease that affects joint linings causing pain, swelling, and deformity (127). The inflamed synovial tissue microenvironment includes an increased number of inflammatory DCs, macrophages and pathological effector T cells. Recent studies have revealed that lactate exacerbates disease severity by regulating migration of immune cells in the arthritic synovium (73, 74). Mainly, lactate inhibits T cell motility, which contributes to their entrapment in the inflammatory site. This depends on the lactate transporters SLC5A12 and SLC16A1 (MCT1) (73, 74). In addition, lactate also drives the differentiation of T helper 17 (Th17) cells that can exacerbate inflammation and disease severity. However, the biological effects of lactate on the APCs under inflammatory conditions are much less understood. Therefore, further investigation requires a more detailed understanding of the lactate effect on different subsets of immune cells under inflammatory versus steady-state conditions.

## Lactate in Regulating Immune Responses to Infections

Emerging studies show that lactate modulates immune responses to infections. As discussed above, DCs and macrophages recognize different pathogens through PRRs, and signaling through these receptors leads to increased glycolysis and increased lactate production. Sepsis is a common and frequently fatal clinical condition characterized by an initial systemic inflammatory response to infection followed by an immunosuppressive phase (128). A recent study utilizing murine models of sepsis has shown that, lactate-GPR81-mediated signaling suppresses the expression of proinflammatory cytokines and induces alternative polarization of macrophages to M2 phenotype (39, 98). Similarly, lactate-induced activation of GPR109a improves survival in mice with sepsis (129). However, the effects of lactate on the functions of DCs and other immune cells in sepsis are unknown. RIG-I-like receptor (RLRs)-mediated signaling is necessary for Type I interferon (IFN) production and this is critical for augmenting host immunity for viral clearance and cancer immune surveillance (10). A recent study has shown that lactate can affect IFN production by negatively regulating the RLR- MAVS-RIG-I pathway (130). Besides, blocking lactate production or metabolism increased type I IFN production with enhanced viral clearance (130). Several pathogens can modulate DC and macrophage function as a mechanism to evade host immune response, resulting in chronic infections such as TB, HIV, HCV, HBV, and SIV (10). However, the role of lactate in the regulation of innate and adaptive immune responses to chronic infections is unknown. In this context, a recent study has shown that in response to *Mycobacterium tuberculosis* (*Mtb*) infection, macrophages switch from pyruvate oxidation to reduction of pyruvate into lactate (131). Besides, Mtb utilizes intracellular lactate as an energy source for growth in macrophages (131). This metabolic switch in macrophages to Mtb infection also increases anti-inflammatory factors such as IL-10 (132). Anti-inflammatory factors produced by APCs play a significant role in establishing chronic infections (10). Collectively, these studies showed that lactate could modulate the immune responses to infections.

## Lactate Signaling in Tumor-Induced Immune Tolerance

Tumors express self-antigens that actively suppress host antitumor immune responses (114, 133). Increased lactate levels positively correlate with tumor grade, progression, recurrence, metastasis, and poor prognosis in several types of cancer (13, 134, 135). As discussed above, lactate secreted by the tumor cells suppresses immune responses by modulating the phenotype and functions of DCs and macrophages in the TME (136, 137). Besides, high lactate levels in the TME impart an anti-inflammatory phenotype on APCs, contributing to immune suppression. Lactate also promotes tumor progression by inducing the prostaglandin E2 (PGE2) synthesis and cyclooxygenase 2 (COX2) upregulation in monocytes (65). PGE2 is a potent immunomodulator that exhibits both proinflammatory and anti-inflammatory effects on DCs and macrophages. Tumors exploit lactate-mediated signaling to effectively suppress host antitumor immune responses (1, 138). DCs and macrophages in the TME express lactate receptors GPR81 and GPR132 (32, 38). The importance of lactate-mediated signaling in controlling antitumor immune responses was demonstrated in a study using GPR81 knockout mice (32). Accordingly, GPR81-deficiency in mice resulted in delayed tumor growth and significantly reduced tumor burden in a syngeneic transplant model and a constitutive breast cancer model in mice (32). Tumor DCs from these mice displayed enhanced activation and increased expression of proinflammatory cytokines such as IL-6 and IL-12 (32). pDCs produce type I IFN and are critical for antitumor immunity. However, pDCs in the TME are dysfunctional and produce low levels of IFNα, which is partly due to lactate in the TME (38). The lactate effect on pDC dysfunction is dependent on GPR81 Signaling and MCTs. Besides, lactate signaling in pDCs induced regulatory T Cell induction by regulating the tryptophan metabolism. Like DCs, macrophages in the TME exert potent effects on cancer metastasis and antitumor immunity. Similar to GPR81, lactate signaling *via* GPR132 in macrophages promotes tumor growth and metastasis (41). Consistent with these observations, mice deficient in GPR132 displayed a significant reduction in tumor burden and breast cancer metastasis. However, the underlying molecular mechanisms are unknown. In addition to APCs, lactate can suppress antitumor immune responses by modulating the functions of other immune cells (67, 70, 71, 83, 84). In summary, these studies reveal an exciting and unappreciated role for lactate in contributing to immune suppression against tumors through different effector mechanisms.

## Targeting the Lactate Signaling Pathway for Immune Modulation and Immunotherapy

There is considerable interest in the lactate signaling pathway as a therapeutic target, especially as a treatment for inflammatory diseases and cancer. Studies involving human cancers and inflammatory diseases strongly suggest that targeting the lactate signaling pathway and lactate metabolism is a promising approach to overcome immune evasion by tumors and suppressing immune-mediated inflammatory diseases. These strategies include targeting signaling (GPR81/GPR132 antagonists), lactate transporters (MCT inhibitors), and lactate metabolism (LDH inhibitors). In addition, pharmacological activators and inhibitors of the lactate signaling pathway exist, and several of them are currently in clinical testing. Here, we will briefly discuss preclinical studies related to the effects of blocking the lactate signaling pathway and lactate metabolism on antitumor immunity and autoimmunity.

## Targeting Lactate-GPR81/GPR132 Signaling

Lactate receptor expression is upregulated in several types of cancer and lactate signaling plays a vital role in tumor development, progression, and metastasis (13, 89, 90). As discussed above, Lactate receptor-mediated signaling in immune cells contributes to the suppression of antitumor immune responses. Thus, blocking specific lactate ligand with cognate GPR81/GPR132 receptors represents a potential strategy to restrain tumor cell proliferation while boosting the antitumor immunity. In this context, a recent study using a 4T1 breast cancer model has shown that intratumoral injection of a GPR81 inhibitor along with an MCT inhibitor resulted in a significant reduction in tumor burden in mice (38). Another critical study has demonstrated that blocking GPR132 signaling in macrophages markedly reduced tumor burden, progression, and breast cancer metastasis in mice (41). Likewise, pharmacological inhibition of GPR132 signaling had a similar effect on tumor burden and antitumor immune responses (139).

On the other hand, activating the GPR81 pathway in APCs may help prevent and treat immune cell-mediated inflammatory diseases such as IBD, hepatitis, and pancreatitis. In this context, our previous study has shown that pharmacological activation of the GPR81 pathway suppressed intestinal inflammation by inducing Treg responses and limiting pathological Th1/Th17 responses. Preclinical studies have shown that lactate treatment suppresses inflammatory responses in the intestine and mitigates intestinal injury (121). Oral administration of lactate had a similar suppressive effect on inflammation-associated gastric injury (140). Likewise, treatment with the GPR81 agonist, 3,5-dihydroxybenzoic acid, ameliorated DSS-induced colitis and reduced inflammation-associated injury in the colon (30). Other studies have shown that pharmacological activation of the GPR81 pathway suppresses inflammation and inflammation-associated tissue injury in other immune cell-mediated inflammatory diseases (31, 141–143). Further studies in understanding the lactate signaling networks in a context-dependent manner will aid in the development of effective treatments for many inflammatory diseases.

## Targeting Lactate Transporters

The second strategy to augment antitumor immune responses involves blocking lactate transporters using MCT inhibitors (22, 144). MCTs are highly expressed in tumors and positively correlate with cancer patients’ poor outcomes (22, 144). Besides, MCTs promote migration and invasion processes in several cancer types, including lung and breast cancers (22, 144). Several studies have examined antitumor immune responses by blocking lactate transport into the APCs using clinically relevant murine tumor models. Blockade of cytosolic transport of lactate in pDCs using AR-C155858 (MCT inhibitor) restored the IFNα production and augmented the immune responses against 4T1 tumors in mice. Furthermore, intratumoral injection of AR-C155858 caused a significant reduction in 4T1 tumor burden in mice (38). Immune checkpoint inhibitors are currently used in cancer immune therapy to enhance immune responses. Another study has shown that silencing MCT1 and MCT4 can restore T cell-induced immune function and boost the immune response to immune checkpoint inhibitors in melanoma patients (75). Also, treatment of Raji xenograft-bearing severe combined immunodeficiency mice with AZD3965 led to inhibition of tumor growth with increased tumor immune cell infiltration involving DCs and natural killer cells (145). MCT1/2 inhibitors are currently in Phase I/II clinical trials to treat patients with advanced prostate cancer, gastric cancer, or diffuse large B‐cell lymphoma (146–150). Collectively these preclinical studies show that drugs that target MCTs alone or in combination with immune checkpoint inhibitors hold much promise as cancer treatments.

In certain autoimmune diseases such as arthritis and MS, MCTs play an inflammatory role. Accumulating evidence shows that blocking lactate efflux or influx has an immunosuppressive effect (151). MCT1/2/4 play a vital role in this, and pharmacological inhibitors of the transporters are attractive targets in these immune-cell mediated inflammatory diseases. Preclinical studies utilizing the murine model of MS have shown that silencing or blocking MCT4 reduced leukocyte infiltration into the CNS and the clinical severity of EAE. Similarly, the silencing of MCT4‐inhibited proliferation of RA synovial fibroblast (RASFs) reduced the severity of arthritis in a mouse model of collagen‐induced arthritis (152). Further studies utilizing murine models of peritonitis and arthritis have shown that blocking or silencing lactate transporter (SLC5A12) restored the T cell functions and ameliorated the disease severity (73, 74, 153). CD147 (EMMPRIN) plays a crucial role in regulating MCT expression by stabilizing and localizing MCTs to the cell membrane. Therefore, disrupting the interaction between CD147 and MCT is also an attractive strategy to regulate immune responses in human diseases. Targeting CD147 has yielded encouraging results in preclinical models of inflammatory diseases (154, 155). Studies have shown that the loss of CD147 function decreases the levels of MCT1 and MCT4 proteins and reduces tumor growth (156–158).

## Targeting Lactate Metabolism

The third strategy to augment antitumor immune responses involves targeting lactate metabolism (23, 159). LDH-A increases the production of lactate in tumor cells and immune cells resulting in tumor immune escape by inhibiting the function of immune cells (71, 160). There is a strong correlation between elevated lactate dehydrogenase (LDH) and poor prognosis in cancer patients. Besides, cancer patients with high LDH levels respond poorly to immunotherapy and other anticancer therapies such as chemotherapy and targeted therapy (159, 161, 162). Thus, targeting the lactate metabolic pathway in immune cells can overcome immune cell dysfunction in the TME. For example, suppressing LDH activity in macrophages can reprogram M2 phenotype to M1 phenotype (163). Besides, deletion of LDH-A in myeloid cells triggers antitumor immunity in the K-Ras murine lung carcinoma model (164). Likewise, blocking LDH in CD8^+^ T cells enhances adoptive T cell therapy (70). Genetic disruption or silencing of LDHA and LDHB in tumor cells inhibits tumor growth (165). Thus, targeting lactate metabolism changes lactate levels in the tumor microenvironment and can enhance antitumor immune responses. These targeting strategies collectively provide attractive angles for immunotherapy but warrant a better understanding of the actions of lactate on immune cells under steady-state and inflammatory conditions.

## Summary

Although lactate was initially recognized as a waste product of cellular metabolism, research over the past decade has revealed a fundamental role for this metabolite in shaping the function of the immune cells. Besides, as evidenced from the discussion above, lactate in the tissue microenvironment programs APCs and other immune cells to regulate the balance between regulatory and inflammatory responses. Though moderate inflammation is essential to mount normal immune responses, uncontrolled, chronic, and excessive inflammation leads to allergic and autoimmune diseases. Lactate exhibits an inflammatory or anti-inflammatory role depending on its effects on immune cell type and disease type. Furthermore, lactate signaling in immune cells could be a critical pathway that links metabolism and immunity. While it is clear that both extracellular and intracellular lactate can program DCs and macrophages to induce robust regulatory immune responses, several important questions remain. For example, how do the lactate signaling pathways regulate adaptive immune responses under homeostatic conditions, inflammation, and cancer?; What are the downstream mediators of the lactate-GPR81/GPR132 pathway?; What role do receptor-dependent and independent lactate signaling play in regulating immunity versus tolerance?; How do the lactate act in concert with other signaling pathways in shaping anti-inflammatory and inflammatory immune responses?; and finally, the question of whether persistent chronic infections such as HIV, HCV or TB exploit the lactate-mediated signaling pathways and, if so, whether blocking this pathway would enhance the immune response is unknown. Addressing these questions will guide the rational design of therapeutic vaccines to reprogram the innate and adaptive immune system towards autoimmune disease tolerance or enhance immune responses against cancer and chronic infections.

## Author Contributions

IM, PP, MT and SM have performed bibliographic researches and drafted the manuscript. All authors contributed to the article and approved the submitted version.

## Funding

We gratefully acknowledge the generous support from Augusta University Intramural Grants Program and the National Institutes of Health (DK123360) for our work.

## Conflict of Interest

The authors declare that the research was conducted in the absence of any commercial or financial relationships that could be construed as a potential conflict of interest.

## Publisher’s Note

All claims expressed in this article are solely those of the authors and do not necessarily represent those of their affiliated organizations, or those of the publisher, the editors and the reviewers. Any product that may be evaluated in this article, or claim that may be made by its manufacturer, is not guaranteed or endorsed by the publisher.
